# EFL learners’ engagement in different activities of blended learning environment

**DOI:** 10.1186/s40862-022-00136-7

**Published:** 2022-05-10

**Authors:** Min Huang, Fangtao Kuang, Ying Ling

**Affiliations:** 1 College of International Studies, Southwest University, Chongqing, China; 2 Faculty of Psychology, Southwest University, Chongqing, China

**Keywords:** Different activities, Learners’ engagement, Blended learning environment

## Abstract

With the blended learning widespread in higher education, the research on the factors which can influence the students’ engagement and their working mechanism has become more and more valuable. In this study, college students’ perception on three aspects, i.e. their cognitive engagement and emotional engagement in two different activities, the activity features and teachers’ roles, has been collected through both questionnaires and interviews. Repeated variance measurement, Pearson correlation analysis and General linear regression have been used to process the quantitative data. Thematic analysis has been used to process the qualitative data. The results show that the cognitive engagement and emotional engagement in Teacher’s Q & A and Online discussion are significantly different (F_CE_ = 10.32**, *ŋ*^*2*^ = 0.07; F_EE_ = 29.60***, *ŋ*^*2*^ = 0.17). Students’ emotional engagement and cognitive engagement in activities are affected by various task features and teacher’s roles. Pedagogical implication and suggestions for further research have also been provided.

## Introduction

With the development of educational technology and the impact of COVID 19, blended learning has gradually become the accepted mode in delivering materials, organizing activities and implementing instruction. According to Garrison and Kanuka (2004), blended learning is the “thoughtful integration of classroom face-to-face learning experience with online learning experience”. Different from the traditional classroom environment, students have two obvious learning channels in blended learning, namely online and offline, and will face new challenges in adapting to this new learning environment and taking greater effort to learn efficiently.

Learner engagement, an important concept in psycho-metrics, has been labeled as the “energy in action”, which is closely related with individual psychological and physical efforts, and characterized by vitality, devotion and focus. Previous research has shown that it is malleable and affected by many factors, such as learning motivation, teaching interference, learning context and teacher-learner relations (Fredricks et al., 2004; Skinner & Pitzer, 2012; Lawson & Lawson, 2013; Xu et al., 2020). In higher education, student engagement is linked with students’ persistence in learning, academic achievement, course satisfaction, graduation rate, etc. (Kuh et al*.*, 2008; Ladd & Dinella, 2009).

Although the blended learning has been a common practice in universities nowadays, the affecting factors and their working mechanism on student engagement have still not been fully explored. In regards to the importance of learner engagement, it is significant to explore the underlying factors on improving the learner engagement in two different learning paradigms of blended learning and provide enlightening suggestions for further teaching practice.

## Literature review

### Blended learning

Blended learning is commonly defined as the combination of traditional face-to-face instruction with online instruction (Bonk & Graham, 2012). It is used in exchange with terms such as integrated, flexible, mixed mode, multi-mode or hybrid learning (Garrison & Kanuka, 2004). Its advantages are apparent, which lies in the combination of merits from both traditional and online approaches. Specifically, the online component of blended learning can allow students to learn anytime and anywhere according to their plan, while classroom activities will provide teacher’s instruction and interaction between peers, which will remedy the absence of group feeling and solve the problem of loneliness in online learning. It is described as a flexible, scalable, active, encouraging, inspiring, active and meaningful way of teaching and learning, and likely to be developed as the leading teaching approach in the twenty-first century (Poon, 2014). Broad view treats blended learning from pedagogical aspect as the combination of different didactic approaches, such as cooperative learning, discovery expository, presentations, and delivery methods, such as personal communication, broadcasting etc. (Graham et al., 2013). When we design and implement BL mode, there exist some challenges. Chen and Yao (2016) use univariate regression analysis to achieve six dimensions which will affect the learners’ satisfaction in blended learning environment, i.e. learner, instructor, course, technology, design and environment. Boelens et al. (2017) proposed four challenges the BL brings with it, namely, incorporating flexibility, stimulating interaction, facilitating students’ learning processes and fostering an affective learning climate. Sahni (2019) categorize the challenges into three aspects of instructors, students and the technical support. Other studies indicate some salient impacting factors, for instance, teachers’ presence in online settings, interactions between students, teachers and content, and deliberate connections between online and offline activities and between campus-related and practice-related activities (Nortvig et al., 2018). All in all, it is highly believed that successful blended learning requires a structured process for designing an effective blend, being rigorous in needs analysis, involving students with appropriate skills and bearing in the mind the organization’s constraints.

Blended learning has also been the focus of ESL/EFL researchers and teachers. Studies show that the blended learning approach can provide many benefits to language learners over traditional teaching approaches, for example, developing language learners’ autonomy, providing more individualized language support, promoting collaborative learning, increasing students’ interaction and engagement, providing opportunities to practice the language beyond the class setting and improving the language skills (Marsh, 2012). Sharma and Barrett (2007) regards that the factors which influence the implementation of blended learning in EFL include teachers’ positive or negative attitudes towards technology use, learners’ proficiency levels, teachers’ training, teachers’ and students’ accessibility to technology, and cost. The related results demonstrate blended learning’s significance in three aspects. First, it is on the relationship between blended learning and students’ performance. For instance, Zhang and Zhu (2018) collect a large database and analyze the students’ variables (gender, grade and knowledge domain), the different learning mode and student performance in both blended learning mode and traditional face-to-face learning mode. The results show that students in blended learning had better academic achievement in their ESL course compared to students in traditional face-to-face learning mode. Ghazizadeh and Fatemipour (2017) design a quasi-experiment to explore the effect of blended learning on the reading proficiency of Iranian EFL learners. The results indicate that the proficiency of reading in blended learning mode is significantly better than that in conventional classroom instruction. Second, it is the students’ attitudes towards blended learning. Akbarov et al. (2018) investigate the students’ attitudes towards blended learning and related concepts. The study revealed that students prefer blended learning to traditional classroom in EFL context. Third, it is on the performance and attitudes. In the study of Banditvilai (2016), blended learning of an English for Specific Purpose (ESP) course in Thailand is focused. The achievements and attitudes in both control group and the experimental group have been compared. The findings show that online practice is directly beneficial to enhance the language skills’ learning as well as autonomous learning and learner motivation. Forth, it is on the learning process and motivation. Liu (2013) studied an Academic English Writing course in blended learning mode. The results show that the students benefit from blended learning, which increases student–student and student–teacher interaction, reduces or even eliminates the communication anxiety, motivates them to become more independent and autonomous learners and enhances their academic writing ability, etc*.* Although the advantages of blended learning in EFL have been proved by enormous studies, the obstacles of designing and implementing blended learning in EFL cannot disappear. All these issues, such as time management, internet access, re-designing the curriculum to meet the students’ need, students’ participation and students’ self-regulation and learning styles, should be carefully considered if higher achievement and richer student learning experience are expected.

### Engagement in blended learning

Helping students engage in learning is an important issue for research in instructional technology (Henrie et al., 2015a, b). Most of the articles reviewed did not have a clear definition statement for engagement (Henrie et al., 2015a, b). Although there has never been a consistent conception on student engagement, researchers agree that engagement is a multi-dimensional and complex concept, which includes two or three distinct sub-constructs, evidenced through a range of indicators. Among them, two common and basic sub-constructs, emotional engagement and cognitive engagement, have been emphasized as crucial and most relevant to blended learning (Halverson & Graham, 2019). Emotional engagement is defined as the emotional response when students are facing the learning tasks, context, peers or instructors, such as belonging, interests, happiness, boredom, etc. It could be both positive and negative reactions. Cognitive engagement is the students’ psychological investment in tasks to comprehend and master content, such as meta-cognitive strategies, preference to challenges, self-regulation skills, etc. (Fredricks et al., 2004; Manwaring et al., 2017). And the crucial factors closely related with study achievement are still emotional engagement and cognitive engagement. It is also the reason why in this study we focus on emotional engagement and cognitive engagement in blended learning environment.

Some factors which could influence the students’ engagement have been found. Hospel and Galand (2016) carries out a study within the framework of self-determination theory and found the main effects and interaction of autonomy support and structure on the different dimensions of engagement. The results also stress the complementary role of autonomy support which is closely associated with emotional engagement. Patall et al. (2016) studied the diaries of high school students in 43 science classes. Multilevel modelling results daily and cumulative interest during class predicted behavioral engagement, cognitive engagement and agentic engagement. Manwaring et al. (2017) applied intensive longitudinal methodology to collect data from students throughout a semester and the results showed that the course design and student perception variables had a greater influence on engagement than individual student characteristics and student multitasking had a strong negative influence on engagement. Students’ perception of the activity importance had a strong positive influence on both cognitive and emotional engagement. Ben-Eliyahu et al. (2018) found that in school settings, self-efficacy was negatively related and mastery goals were positively related to affective engagement and overall engagement predicted all forms of motivation. From these studies, we can summarize the factors into extravert and introvert aspects. The former one focuses on the students’ learning characteristics, such as their interests, motivation, self-efficacy on engagement. The latter one concentrates on the course design, teachers’ roles and technical support.

Further, measurement of student engagement is crucial because design of instruction needs good evaluation of the efficacy of instructional intervention. However, students’ engagement is considered to be malleable and it is not stable across learning situations and school subjects, its measurement is always be the difficult point for researchers. The literature review shows that most of existing studies use quantitative self-report, for example, surveys, scales, or questionnaire. Other methods include qualitative measure (such as interview and discourse analysis), quantitative observational measures (such as number of assignments turned in) and bio-physiological sensors (such as eyes movement tracking) (Henrie et al., 2015a, b). Among these measure methods, each method has its own advantages and disadvantages. For example, quantitative self-report can be easily to be distributed, useful for self-perception and less observable engagement indicators, but may be tedious if frequent repeated measures are necessary and it could be intrusive for the class time. And interview can enable the data gathering without disrupting learning, but it is difficult to scale it. Thus, mixed research methods which could combine advantages from different methods are suggested by some researchers, in particular for engagement research in blended learning where two learning channels exist. In this study, we will use both questionnaires and interviews to collect data. Many instruments have been developed to investigate the students’ engagement on school and course level, but little has been done on the activity-level. Sinatra et al. (2015) recommend that the instrumentation should be considered on a grain-size continuum, ranging from a person-centered to a context-centered orientation for clarifying measurement issues. Because of the inherit characteristics of malleability of engagement, it is important to gain an insight into the real-time engagement from students at activity level, which is the direct motivation for this study.

Blended learning method and students’ engagement have closely relationships. Researchers propose that one reason why blended learning is becoming popular is the perception that blended learning can increase student engagement (Garrison & Kanuka, 2004). More and more studies focus on student engagement in blended learning environment. Delialioğlu (2012) designed, implemented and compared a lecture-based blended learning and a problem-based blended learning. Repeated measure ANOVA analysis on data revealed that the indicators of student engagement were significantly higher in the problem-based blended learning than that in lecture-based blended learning. Regression analysis showed that the difference in active learning is due to the learning environment provided in the problem-based blended learning. Henrie et al. (2015a, b) collected both self-report and observational data to explore the change of engagement over time at the course level and between students and identify patterns and influences of student engagement in a blended course. The results indicate that clarity of instruction and relevance of activities influenced student satisfaction more than the medium of instruction. Sahni (2019) collect such multiple source data as focus-group interviews, student surveys and LMS records. The results show that the students in blended learning group outperform in the aspects of outcome achievement and overall engagement with online activities as well as in class activities. Halverson and Graham (2019) present a framework for engagement in blended learning environment. The factors influencing the cognitive engagement cover attention, effort, time on task, cog/meta-cognitive strategies, deep concentration/absorption, or individual interest and curiosity. For emotional engagement, it covers enjoyment, happiness and confidence for positive side, and boredom, frustration and anxiety for negative side, and confusion between positive side and negative side. Li et al. (2021) prove that face-to-face class attendance has positive correlation with virtual learning engagement and the face-to-face class attendance and virtual learning engagement both have positive correlation with academic performance.

Although studies prove that engagement is crucial for students to gain new skills and knowledge, however, it has always been problems in blended learning implementation (Ma’arop & Embi, 2016). Two factors should be in focus, i.e. the characteristics of learners and the learning platform or social medium devices. Tay (2016) found that designers of blended professional development courses should bear in mind the characteristics of both the learner and the online platform to achieve greater cognitive, behavioral and social engagement. Bo et al. (2020) use the questionnaire to examine the relationship between students’ perception of blended learning platform and course satisfaction based on engagement. Their results indicate that satisfaction in blended learning mode is influenced by emotional engagement and perceived playfulness of blended learning platform. Meanwhile, platform’s perceived usefulness has a stronger direct influence on students’ cognitive and emotional engagement in blended learning. Bedenlier et al. (2020) reviewed 42 peer-received arts and humanities articles published from 2007 to 2016. The results indicate that educational technology in blended learning supports student engagement. Blogs, mobile learning and assessment tools were the most effective at promoting engagement.

From the previous studies, we can summarize that the researchers’ interest of engagement in blended learning is on the course level, exploring the actors which will influence the level of engagement. However, the activities are the core element in a course and the engagement, as a situation property, will change according to the context. It is significant to explore the factors on activity level which will affect students’ engagement and provide meaningful suggestions for efficient activity design.

## Research design

### Research questions

Based on the significance and necessity mentioned previously, this study aims to investigate the learners’ perception of engagement in different activities and their correlation with activity features and teacher’s roles during implementation in blended learning. Specifically, three research questions have been raised as follows.

- Question 1: Is there a significant difference between students' cognitive engagement in Teacher’s Q & A and that in Online asynchronous discussion? If yes, how?
- Question 2: Is there a significant difference between students' emotional engagement  in Teacher’s Q & A and that in Online asynchronous discussion? If yes, how?
- Question 3: What are the underlying factors that contribute to the emotional engagement and cognitive engagement in these activities?

Among them, Teacher’s Q & A is a classroom activity, in which the teacher will organize the students’ translating the English text into Chinese by the reason that the teacher believe the translation method is still the efficient way for Chinese students to comprehend the text deeply. The students will voluntarily ask for the chance to translate the paragraph after they think they have prepared well. Then, the teacher will ask related questions for students to answer. In this way, they can further infer the indicated meanings between lines. Their performance will be recorded for course’s procedural evaluation. Online discussion is taken in QQ group where all students have entered in. The teacher will post the discussion topics related to the content of the unit and set the deadline time for the students to express their ideas freely.

### Data collection

A mixed method, which consists of quantitative and qualitative ways of collecting and analyzing data, was operated in this study. To collect quantitative data, we used a questionnaire, once used by Henrie et al. (2015a, b), which was proved to be with high reliability and construction validity and appropriate to collect data on the activity level. To collect qualitative data, interviews with students after the class were used. From the quantitative data, we can understand if there is statistical difference between emotional engagement and cognitive engagement in different activities and what factors can contribute to the learners’ engagement in these two activities. From the qualitative data, we can dig deeply on the students’ perception on activities and their specific understanding of the conceptions of the potential reasons.

Meanwhile, a blended English reading course was redesigned and constructed in the university where the authors work. The teacher has nearly 20 years’ of teaching experience, and she is skillful in solving the problems in teaching and meeting the challenge of new demands. She also participated the training of modern teaching technology and learned blended teaching theories to guarantee the sound teaching practice. The framework of this course was set up around the following elements, namely, resource, activities, students, teachers, and evaluation. To scaffold rich learning resources, teaching videos explaining the meanings and usage of new words and difficult sentences in reading text were recorded in advance by the teacher and post on the Superstar learning platform, which is popular in China and convenient for students to access to. Students are required to watch the videos at set time. Meanwhile, QQ groups, a widespread chatting app in China, were established, in which teaching notices and online discussion topics are delivered. Learning activities, such as teacher’ Q & A and students’ demonstration, were done in class time and watching teaching videos and topic discussion were taken on such digital channels as superstar learning platform or QQ group. To prepare the students in this course, we inform them the course aim and teaching procedures at the beginning of the course.

### Participants

146 university students have voluntarily participated in this study. They are sophomore, tested as intermediate level of students before the class. They selected this English reading course by their own and most of them want to improve their reading proficiency. Among them, 49.31% (n = 72) are male students and 50.69% (n = 74) are female students. 31.51% of them (n = 46) major in Liberal arts, 41.78% (n = 61) Science, and 26.71% of them (n = 39) major in Engineering. 61.64% (n = 90) have more than one month of blended learning experience and 38.36% (n = 56) have no more than one month learning. 26.03% (n = 38) accept the blended learning completely. 37.67% (n = 55) somewhat accept. 32.88% (n = 48) feel it doesn’t matter. 2.74% (n = 4) feel it a little unacceptable. 0.68% (n = 1) does not accept it completely. They were informed by the researchers that they would participant in a program and questionnaires would be done on their own content.

### Instruments

The questionnaire, proposed and validated by Henrie et al. (2015a, b), consists of seven items on the aspects of emotional engagement and cognitive engagement. Emotional engagement covers the indicators of enjoyment, excitement, interest and preference during a specific learning activity. Cognitive engagement covers indicators of concentration, passive/active, and focus/distract. The questionnaires were delivered to students through Wenjuanxing app platform on the fifth academic week just after the teacher’s Q & A activity in classroom and students’ discussion in QQ group. The Cronbach’*a* for Teacher’s Q & A questionnaires is processed to be 0.89 and the Cronbach’*a* for online discussion questionnaires is 0.91. This means the questionnaire has a high reliability.

The interview questions were elicited according to the questionnaire results and designed for deeper and specific information about students’ perception of learning engagement. 1) What can make you think Teacher’s Q & A and Online discussion is interactive? 2) What can make you think Teacher’s Q & A and Online discussion is valuable? 3) What can make you think Teacher’s Q &A and Online discussion facilitate the autonomy in you? Eight students voluntarily participated in the interview. Their responses were recorded and later transcribed into word format.

## Results and discussion

The data collected by questionnaires were processed by method of Repeated measure ANOVA in SPSS 30. The results are shown as follows (Table 1).

**Table 1:** Results 1 of repeated measure ANOVA (n = 146)

Variable	Teacher’s Q & A		Online discussion		F(*p*)	*ŋ*^*2*^
	Mean	SD	Mean	SD		
Emotional engagement	3.64	0.42	3.39	0.61	29.60***	0.17
Cognitive engagement	3.55	0.56	3.37	0.72	10.32**	0.07

*p < .05; **p < .01; ***p < .001

The results show the emotional engagement in Teacher’s Q & A and Online discussion is significantly different (F = 29.60***, *ŋ*^*2*^ = 0.17). The level of emotional engagement in Teacher’s Q & A is higher than that in Online discussion (M_T_ = 3.64 > M_O_ = 3.39). The cognitive engagement in Teacher’s Q & A and Online discussion is also significantly different (F = 10.32**, *ŋ*^*2*^ = 0.07). The cognitive engagement level in Teacher’s Q & A is also higher than that in Online discussion (M_T_ = 3.55 > M_O_ = 3.37).

Table 2 indicates that the task interactivity in Teacher’s Q & A and Online discussion is significantly different, with the former one higher than the latter one (F = 95.93***, *ŋ*^*2*^ = 0.40). There is no significant difference between students’ perceptions on activities’ challenge, relatedness, values and autonomy. The interview with students implies that the interactivity in the Teacher’s Q & A activity lies in that the teacher usually asked the students to answer her questions. In this way, they think the interactivity is stronger than that in Online discussion although they would still discuss the topics in QQ group with each other. The reason why the students think the interactivity in Online discussion is relatively low might be that the teacher’s absence from the discussion task did not provoke the students’ interest in exchanging their ideas. As Chinese students, without teachers’ instruction, they prefer to read others’ ideas and do not comment on. So, a better design for Online discussion could involve the presence of discussion tutors, who could be activity organizers or the teacher.

**Table 2:** Result 2 of repeated measure ANOVA (n = 146)

Variables	Teacher’s Q & A		Online discussion		F(p)	*ŋ*^*2*^
	Mean	SD	Mean	SD		
Challenge	3.47	0.66	3.45	0.67	0.09	0.00
Relatedness	3.50	0.79	3.62	0.84	2.44	0.02
Value	3.92	0.56	3.86	0.69	1.18	0.01
Interactivity	3.82	0.63	3.1781	0.84	95.93***	0.40
Autonomy	3.51	0.76	3.4041	0.78	1.94	0.01

^*^p < .05; **p < .01; ***p < .001

Results from Table 3 show that the teacher’s notification and encouragement in both Teacher’s Q & A and Online discussion have no significant difference (F1 = 3.34, *ŋ*^*2*^ = 0.02; F2 = 1.46, *ŋ*^*2*^ = 0.01). However, the teacher’s instruction in Teacher’s Q & A is significantly different from that in Online discussion (F = 19.89***, *ŋ*^*2*^ = 0.12). It conforms with the teaching practice in which the teacher will instruct more in Teacher’s Q & A than that in Online discussion. Because Teacher’s Q & A is a face-to-face activity, the teacher could act better as an instructor than in Online discussion where teacher’s presence is lack somewhat. And teacher’s feedback in Teacher’s Q & A is significantly different from that in Online discussion. The feedback in Teacher’s Q & A is higher than that in Online discussion. In classroom, the teacher could give the feedback directly and timely after students answer the questions while Online discussion cannot make the feedback instant and the frequency of feedback is not so high, neither. These differences are determined by the activities’ inherit features.

**Table 3:** Result 3 of repeated measure ANOVA (n = 146)

Variable	Teacher’s Q & A		Online discussion		F(*p*)	*ŋ*^*2*^
	Mean	SD	Mean	SD		
Notification	4.11	0.61	4.22	0.73	3.34	0.02
Instruction	4.03	0.61	3.73	0.80	19.89***	0.12
Encouragement	3.95	0.68	3.88	0.76	1.46	0.01
Feedback	4.05	0.52	3.83	0.81	12.64**	0.08

*p < .05; **p < .01; ***p < .001

Table 4 indicates the correlation between different variables in both Teacher’s Q & A activity and Online discussion. It shows that the task features (challenge, relatedness, value, interactivity, autonomy) are all related with students’ emotional engagement and cognitive engagement in both activities. Meanwhile, teacher’s roles (notification, instruction, encouragement, and feedback) are all related with students’ emotional engagement and cognitive engagement. In this way, further analysis can be done.

**Table 4:** Pearson correlation analysis (n = 146)

	1	2	3	4	5	6	7	8	9	10	11
1. Emotion	–	.50***	.45***	.48***	.55***	.56***	.41***	.41***	.46***	.46***	.54***
2. Cognition	.69***	–	.35***	.42***	.47***	.48***	.52***	.42***	.42***	.36***	.36***
3. Challenge	.29***	.28**	–	.49***	.32***	.36***	.406***	.20*	.24**	.28**	.27**
4. Relatedness	.47***	.46***	.34***	–	.58***	.35***	.37***	.28***	.33***	.37***	.38***
5. Value	.50***	.52***	.34***	.60***	–	.49***	.31***	.45***	.52***	.44***	.48***
6. Interactivity	.47***	.40***	.29***	.35***	.41***	–	.46***	.47***	.45***	.46***	.43***
7. Autonomy	.40***	.55***	.30***	.38***	.40***	.39***	–	.24**	.21**	.24**	.28***
8. Notification	.22**	.26**	.16*	.27**	.39***	0.16*	.21*	–	.73***	.63***	.57***
9. Instruction	.31***	.31***	.32***	.28**	.34***	.45***	.31***	.54***	–	.66***	.64***
10. Encouragement	.33***	.30***	.29***	.33***	.40***	.42***	.30***	.50***	.74***	–	.61***
11. Feedback	.33***	.30***	.33***	.33***	.37***	.48***	.40***	.38***	.76***	.75***	**–**

*p < .05; **p < .01; ***p < .001. The right upper half is the correlation between engagement and task features and teacher’s roles in Teacher’s Q & A activity and the left lower half is the correlation between engagement and task features and teacher’s roles in online discussion activity

From Table 5 we can notice that students’ emotional engagement in Teacher’s Q & A can be explained by such four variables as interactivity, teacher’s feedback, challenge and value in sequence. Students’ cognitive engagement in Teacher’s Q & A can be explained by just one variable: autonomy. Interviews with the students show that the teacher usually ask students translate the paragraphs of the reading text in the classroom and then asked related questions to facilitate the deep understanding of the learning content, which makes them think Teacher’s Q & A is a challenging work. Because they want to translate the paragraphs in the right way, they must manage to know the meaning of words and phrase and organize the logical Chinese sentences, which are syntactically different from English ones. But they also think that before answering the teacher’s questions, they must first consult the dictionaries for the words’ meaning and organize their language for fluent translation, which is valuable for their autonomous study. Meanwhile, the topics they studied, such as college life, emotions, sporting life, are valuable for their later life and work. They think the interactivity between the teacher is a happy experience because even if they answered the questions in a wrong way, the teacher did not criticize them severely and their self-esteem was not been damaged. It shows the importance of teacher’s feedback skills. And they can voluntarily answer the questions, which makes them think they have the learning autonomy.

**Table 5:** Result 1 of Multiple linear regression analysis (n = 146)

	Teacher’s Q & A							
	Emotion engagement				Cognitive engagement			
	B	SE	*β*	T(*p*)	B	SE	*β*	T(*p*)
constant	0.99	0.22		4.34***	0.68	0.32		2.13*
Challenge	0.10	0.04	0.16	2.33*	0.02	0.06	0.033	0.44
Relatedness	0.04	0.04	0.08	1.05	0.07	0.06	0.106	1.25
Value	0.14	0.06	0.19	2.30*	0.13	0.08	0.139	1.58
Interactivity	0.16	0.05	0.24	3.11**	0.10	0.07	0.119	1.44
Autonomy	0.04	0.03	0.08	1.10	0.24	0.05	0.33	4.44**
Notification	− 0.01	0.06	− 0.02	− 0.24	0.12	0.08	0.135	1.39
Instruction	0.01	0.06	0.01	0.12	0.11	0.09	0.123	1.16
Encouragement	0.02	0.05	0.04	0.50	− 0.01	0.07	− 0.021	− 0.22
Feedback	0.18	0.06	0.23	2.74**	− 0.04	0.09	− 0.038	− 0.43
F(*p*)	17.25***				12.55***			
*R*^*2*^	0.52				0.44			

*p < .05; **p < .01; ***p < .001

Table 6 indicates that students’ emotional engagement in Online discussion is influenced by interactivity, value and relatedness in sequence. And students’ cognitive engagement in Online discussion is influenced by autonomy and value. The interview with the students shows that in Online discussion activity, the topics they discussed are related to their life. For example, one topic is about school bullying. A student reflected that because he had the similar experience when in middle school, he had a strong desire to express his idea. And Online discussion activity can help them to practice their writing skills because they must express their ideas in written words instead of sound, which made them think the Online discussion is valuable besides that they can share their ideas and read others’. Further, they can determine the time to participate in the discussion and have the right to express their ideas freely, which makes them think they have the learning autonomy.

**Table 6:** Result 2 of Multiple linear regression analysis (n = 146)

	Online discussion							
	Emotion				Cognitive			
	B	SE	*β*	T(*p*)	B	SE	*β*	T(*p*)
Constant	1.09	0.32		3.38**	0.44	0.36		1.21
Challenge	0.03	0.06	0.04	0.49	0.02	0.07	0.02	0.27
Relatedness	0.13	0.06	0.19	2.15*	0.10	0.07	0.13	1.53
Value	0.18	0.08	0.21	2.29*	0.25	0.09	0.25	2.81**
Interactivity	0.18	0.06	0.25	2.99**	0.10	0.06	0.12	1.45
Autonomy	0.10	0.06	0.13	1.67	0.33	0.06	0.36	4.78***
Notification	− 0.01	0.07	− 0.02	− 0.21	0.02	0.08	0.03	0.31
Instruction	0.03	0.09	0.04	0.36	0.09	0.10	0.11	0.93
Encouragement	0.05	0.09	0.06	0.56	0.01	0.10	0.01	0.11
Feedback	− 0.05	0.09	− 0.08	− 0.65	− 0.12	0.10	− 0.15	− 1.25
F(*p*)	9.40***				11.90***			
*R*^*2*^	0.38				0.44			

*p<.05; **p<.01; ***p<.001

## Conclusion

In regards to the tremendous advantages of blended learning, many courses in higher education have changed their delivery method. It has transferred the emphasis of education from teaching to learning, thus enhancing the students’ learning experience and, consequently improves their course satisfaction and academic results. Further, activity-level measures enables us to identify relationships between types of learning activities and types of engagement (Henrie et al., 2015a, b) In this study, we found that the emotional engagement and cognitive engagement in Teacher’s Q & A are significantly different from those in Online discussion. Further, we found that task interactivity and value can account for emotional engagement both in Teacher’s Q & A and Online discussion. And task autonomy can explain the cognitive engagement in both Teacher’s Q & A and Online discussion. Thus, some meaningful and practical pedagogical suggestions have been attained to design efficient activities both in classrooms and online in blended learning course to achieve high level of students’ engagement.

First, activities in blended learning environment should facilitate the students’ interaction with instructors and peers. According to constructivist theory, meaning occurs in the individual and results from his or her experience and social interaction with others (Dawley, 2007). Meaningful learning occurs when individuals are engaged in social activities, i.e. interacting with one another. From the interactionist perspective, interaction in second language learning was viewed as the context and process through which language can be learned. And three theoretical perspectives were involved, i.e. as interaction of learner needs (need to understand the target language and express it across modality with accuracy and appropriately); interaction of learning processes (cognitive, psycholinguistics, social); and interaction of learner with native speaker interlocutors and with other others (Pica, 1998). Blended learning modality asks for the interaction between learners and learning materials, learning context, and other agents. From the results of this study, we noticed that when the activities are designed to facilitate the occurrence of interaction between students and teachers (such as in Teacher’s Q & A), students and learning materials or other students (such as in Online discussion), learners’ emotional engagement will be increased, which will ultimately be expected to increase the learning satisfaction. The essence of interaction is to make the information shared and flow between different learning agents and make the knowledge learned in this process.

Secondly, activities in blended learning environment should bear the property of task value. Task value was defined as the learners’ perceptions of the interest, usefulness, importance and cost of a task. It is the factor to predict learners’ decision to further follow the task or not (Wigfield & Eccles, 2000). In other words, if students think that a task is in high value, they are more likely to participate in the activity, employ more efficient cognitive strategies, persist for a longer time and devote further efforts in it. Task value is considered as a meaningful determiner of learners’ language achievement (Joo et al., 2013). The decision-making process will help to assess the subjective value of a task. In this process, the students will consider the importance of a specific task, the personal interest of the task, its usefulness in relation to future personal goals, and the cost or the perceived negative aspects of engaging in this task (Metallidou & Vlachou, 2007). In this study, we found that the students’ cognitive engagement in both activities will be affected by their perceptions of task value, which is accordance with previous study, which proposes that high task value will activate students’ effort and time and consequently, their cognitive engagement through the application of deep cognitive and meta-cognitive strategies (Wigfield & Eccles, 2000). The interviews of the students indicate that the students in English blended course will think such activities as valuable: i) the activities which can facilitate their language proficiency, such as translation skill or writing ability; ii) the activities which can enrich their general knowledge, for example, the various ideas from others, which they don’t know before; iii) the activities which can facilitate their social skills, such as fluent expression of their ideas in public circumstances.

Thirdly, activities in blended learning environment should involve and facilitate the learners’ autonomy. Good language learners should be autonomous ones, who can set their learning goals, select learning methods and techniques, monitor the learning speed, and evaluate the learning process. Further, they are able to solve the problems through independent thinking and learning. In short, Holec (1981) defined the learner autonomy, which is the ability to take charge of one’s own learning. However, learners do not automatically accept responsibility for their learning and they will not necessarily find it easy to reflect critically on the learning process. Thus, teachers should design the activities to promote the learner autonomy, in this way to lead to high level of students’ engagement in learning. Three aspects could be considered when we are designing the activities to involve the learner autonomy, i.e. technical, psychological, social-cultural (Oxford, 2003). In blended learning, technical perspective allows us to comprehend the functions of modern technology and social medium in information conveying and delivery. Psychological perspective asks for the focus on students’ desire to seek meaning, positive attitudes, need for achievement and an intrinsic and extrinsic motivation. Social cultural perspective requires teachers regard students as self-regulated persons who will develop through interaction with more capable, mediating person in a particular context. For the online activities in blended learning course, the application of social mediums enables the students communicate with teachers and classmates at any time and any place, which the students can determine according to their situation. Specifically, in the Online discussion activity, after the topic has been delivered to the students, they can first make an independent thinking on how to respond to the discussed topic, then they can come to website to expand their related knowledge and form their own ideas. Next, they can decide when they can post their ideas in QQ group. The students take control of all the learning process and are responsible for their learning. For the face-to-face classroom activities in blended learning, such as in Teachers’ Q & A, students could first autonomously study the paragraphs in the text and translate them into Chinese. Then, they will be responsible for determining whether they will take the chance to answer the teachers’ questions or not. The principle of designing activities to promote autonomy is to make students realize that they are responsible for the accomplishment of the activity, which is considered as the important issue in developing learners’ autonomy (Wenden, 1991).

In this study, we also find the importance of teacher’s role in affecting the emotional engagement when implementing Teacher’s Q & A activity, specifically the teacher’s feedback, which has a powerful influence on accelerating students’ learning, where the teacher plays an important role in directing students’ attention to learning goals and criteria (Andrade & Brookhart, 2016). EFL learning involves subject-specific challenges for students. Students’ depression and errors in the process is the common existence. Teacher’s feedback can help equip students with correct knowledge or personal encouragement, in this way to encourage thinking, motivate and promote learning. Meanwhile, students’ confidence will be re-established. The interviews with the students indicate that teachers’ attitudes and kind response towards their mistakes and teachers’ constant oral encouragement make them feel relaxed and they refer to continue studying even if they frequently make mistakes.

## Data Availability

Attached Excel file named Raw Data.

## Abbreviations

EFL: English Foreign Language
ESL: English Second Language
Teacher’s Q & A: Teacher’s Questioning and Answering
CE: Cognitive Engagement
EE: Emotional Engagement
QQ: “Open I Seek You” software
M_T_: Mean score of Teacher’s Q & A
M_O_: Mean score of Online Discussion
et al*.*: And other people
i.e.: That is
